# Future Prospects of Positron Emission Tomography–Magnetic Resonance Imaging Hybrid Systems and Applications in Psychiatric Disorders

**DOI:** 10.3390/ph15050583

**Published:** 2022-05-08

**Authors:** Young-Don Son, Young-Bo Kim, Jong-Hoon Kim, Jeong-Hee Kim, Dae-Hyuk Kwon, Haigun Lee, Zang-Hee Cho

**Affiliations:** 1 Department of Biomedical Engineering, College of Health Science, Gachon University, Incheon 21936, Korea; ydson@gachon.ac.kr; 2 Neuroscience Research Institute, Gachon University, Incheon 21565, Korea; neurokim@gachon.ac.kr (Y.-B.K.); jhnp@chol.com (J.-H.K.); 3 Department of Neurosurgery, Gachon University College of Medicine, Gil Medical Center, Gachon University, Incheon 21565, Korea; 4 Department of Psychiatry, Gachon University College of Medicine, Gil Medical Center, Gachon University, Incheon 21565, Korea; 5 Biomedical Engineering Research Center, Gachon University, Incheon 21936, Korea; jhkim1104@gachon.ac.kr; 6 Neuroscience Convergence Center, Institute of Green Manufacturing Technology, Korea University, Seoul 02841, Korea; davidkwon2501@gmail.com; 7 Department of Materials Science and Engineering, Institute of Green Manufacturing Technology, Korea University, Seoul 02841, Korea

**Keywords:** positron emission tomography, magnetic resonance imaging, high resolution, hybrid imaging, psychiatric disorders

## Abstract

A positron emission tomography (PET)–magnetic resonance imaging (MRI) hybrid system has been developed to improve the accuracy of molecular imaging with structural imaging. However, the mismatch in spatial resolution between the two systems hinders the use of the hybrid system. As the magnetic field of the MRI increased up to 7.0 tesla in the commercial system, the performance of the MRI system largely improved. Several technical attempts in terms of the detector and the software used with the PET were made to improve the performance. As a result, the high resolution of the PET–MRI fusion system enables quantitation of metabolism and molecular information in the small substructures of the brainstem, hippocampus, and thalamus. Many studies on psychiatric disorders, which are difficult to diagnose with medical imaging, have been accomplished using various radioligands, but only a few studies have been conducted using the PET–MRI fusion system. To increase the clinical usefulness of medical imaging in psychiatric disorders, a high-resolution PET–MRI fusion system can play a key role by providing important information on both molecular and structural aspects in the fine structures of the brain. The development of high-resolution PET–MR systems and their potential roles in clinical studies of psychiatric disorders were reviewed as prospective views in future diagnostics.

## 1. Introduction

The brain, the most complex organ in the human body, plays an important role in modulating physiological responses, including human behaviors and cognition. Modern imaging techniques include computed tomography (CT) [1], magnetic resonance imaging (MRI) [2], and positron emission tomography (PET) [3,4]. These medical imaging devices provide structural, functional, and molecular information regarding the human body and brain. This information is visualized in image formats or quantitated numeric values. The PET–CT system was one of the first successful hybrid models, successfully providing high-resolution anatomical and molecular images and simultaneous functional and molecular information [5]. Many clinicians expected that the PET–MRI fusion images could be more useful in the brain application rather than PET–CT fusion images because MRI can provide higher contrast between gray matter and white matter in the brain than CT [6]. However, the PET–MRI fusion system is not yet considered a successful hybrid system compared to the PET–CT system due to the various technical challenges of combining PET and MRI. In particular, because the brain is compactly populated with very small areas, if the spatial resolution of PET and MRI does not match in the PET–MRI fusion, molecular information irrelevant to the structural location is provided, making the accuracy of quantitative analysis unreliable. Therefore, matching the spatial resolution between the two images in PET–MRI fusion imaging of the brain is important. In terms of spatial resolution, the current performance of PET cannot meet the anatomical discrimination requirements of the brain. Furthermore, it is imperative to improve the spatial resolution of the PET system, possibly to approximate that of the MRI system, which has a much better resolution.

Unlike brain tumors, where the anatomical features of lesions are distinct, many mental disorders require functional and molecular imaging with the high anatomical resolution because they require precise anatomical locations and information, especially for the treatment of psychiatric disorders. In summary, improving the resolution and specificity performance of imaging technologies is crucial for clinical pharmaceutical medicine. This study will briefly review the advancements in MRI and PET imaging technology, especially the newly developed PET–MRI fusion molecular imaging system and its applications.

## 2. MRI-PET Fusion Imaging

### 2.1. MRI

**Figure 1:**
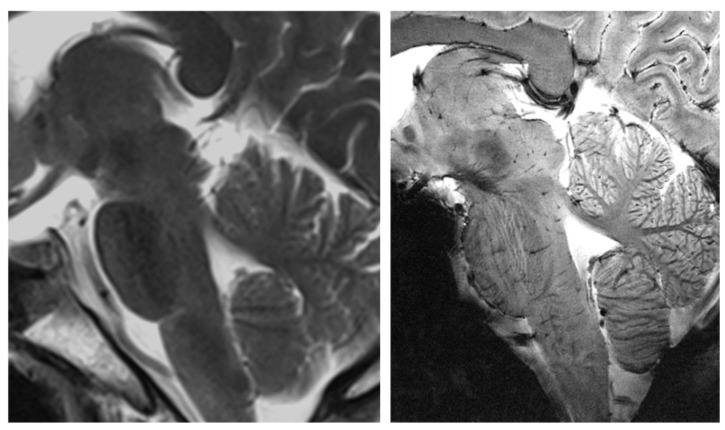
Comparison of 3.0T (**left**) and 7.0T (**right**) MRI in the brainstem area. As shown above, markedly different detail is visualized on the 7.0T MRI, such as the details of the thalamic and brainstem areas, including the midbrain and pons. Reprinted with permission from Cho et al., 2014, Elsevier [13].

MRI in recent decades has progressed mainly due to the introduction of ultra-high field (UHF) MRI. Currently, the widely used field strength of MRI for clinical use is 1.5 and 3.0 tesla (T) together with UHF, such as 7.0T MRI for research. On 12 October 2017, the United States Food and Drug Administration approved 7.0T MRI for clinical use. Images obtained from 7.0T MRI showed markedly improved images with a high signal-to-noise ratio (SNR) and other imaging modalities such as tractography [7,8]. This improvement has enabled the visualization of many structures hitherto unavailable in lower-magnetic-field systems [9,10,11,12]. This was particularly obvious in areas that require high spatial resolution, such as the brainstem, hippocampus, and thalamus, which have complicated substructures. Figure 1 shows an example of the brainstem area, which is composed of various small subnuclei, in 3.0T and 7.0T MRI [13].

Depending on the region of interest (ROI), different MRI modalities can be selected. For imaging the subnuclei of the thalamus, T1-weighted MRI with inversion recovery appears to be more suitable for maximizing the contrast of the subnuclei by nullifying the signal of a specific nucleus [14,15,16,17]. T2*-weighted imaging with a gradient echo pulse sequence can achieve better contrast between the white and gray matter. Further discrimination of white and gray matter can also be delineated using other techniques, such as diffusion-weighted imaging [18,19,20].

With an increase in the magnetic field, a high SNR and spatial resolution were achieved, as well as the uniformity and linearity of the magnetic field. However, in a high field such as susceptibility, it is higher than in a lower field. These magnetic field disturbances often result in image distortion and the mislocation of anatomical structures. The uniformity problem is related to the uniformity of the main magnetic field and the local linearity of the gradient magnetic field generated by the gradient coil. These issues can be particularly highlighted and challenged in the development of PET–MRI systems in which the PET module is inserted and integrated into the bore of the MRI [21].

A higher gradient field is also required to achieve a higher spatial resolution. However, this leads to a lower SNR and longer acquisition time due to the decrease in voxel size and the increased number of voxels. To increase the SNR under a given magnetic field, the development of radiofrequency (RF) coil technology, such as a multichannel phased-array coil, is also important. The sensitivity of the MR signal can be increased by optimizing the transmission and reception of the RF coil system [22]. The multichannel transmission/reception RF coil facilitates the reduction of the acquisition time by combining various recently introduced data sampling strategies, including compressed sensing technology.

### 2.2. PET

The PET system was designed to provide functional and molecular imaging of human organs, including the brain. This information depends on the properties of the radiopharmaceuticals used for PET imaging. Radiopharmaceuticals are synthesized using positron-emitting radionuclides and chemical compounds with an affinity for target molecules. The emitted positron is annihilated by an electron, and two gamma photons are generated. The PET system collects gamma photons for position determination using detectors (scintillators) with high atomic numbers. Photomultiplier tubes (PMTs) and silicon photomultipliers (SiPMs) are the two main devices used to convert scintillation light into a detectable electrical signal. SiPMs have a high gain similar to that revealed by PMTs and resistance to the high magnetic field, making them an acceptable alternative to PMTs in the PET–MRI system [23]. The new digital SiPMs provide good timing, energy, spatial resolution, and temperature stability, making them a promising candidate for MR compatibility [24,25,26]. After the signals are digitized, the pulses and addresses encoding the position of the detectors are processed to generate list-mode data containing pulse arrival time, energy, and position information.

Although PET typically provides sensitivity in the nM to pM range, the spatial resolution of PET images is hampered by the physical properties of detectors and gamma photons [27,28], such as detector size, positron range [29,30], penetration effect, and non-collinearity. In PET, the effect of these physical factors appears as a blurring of the image. To improve the spatial resolution, technical developments have been made in the PET detector system, particularly a decrease in the detector size to improve the intrinsic resolution of the system. Most PET systems designed today have the smallest detector size possible, but this approach reduces detection efficiency and increases the penetration effects on adjacent detectors, thereby limiting the further reduction in detector size [31,32]. The depth of interaction (DOI) design of the detector can improve the spatial resolution by decreasing the penetration effect. However, it is often impractical to employ in a real-world system.

**Figure 2:**
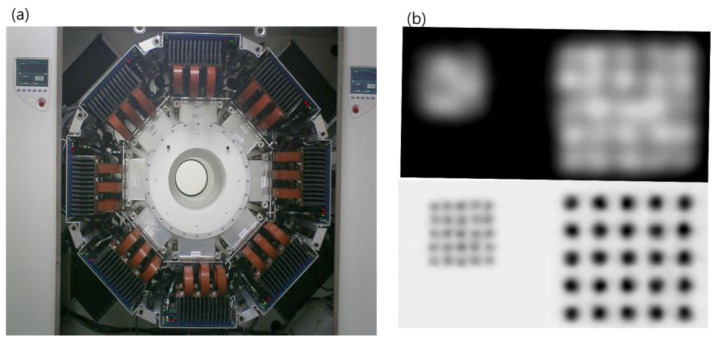
(**a**) HRRT-PET system. (**b**) The resolution of the phantom image obtained from conventional PET–CT (upper) and HRRT-PET (lower) with a 2 mm diameter radioisotope bar separated by 4 mm. Conventional PET–CT has a resolution of <5 mm FWHM [34], while HRRT images have a resolution of 2.5 mm FWHM.

High-resolution research tomograph (HRRT) (Siemens Healthcare, USA) is a leading high-resolution PET system among many brain-dedicated PET systems [33]. Unlike other commercial PET systems, the HRRT was designed for research purposes with 119,808 detectors, each of which has a width as small as 2.3 mm. Dual-layer lutetium oxyorthosilicate and lutetium-yttrium oxyorthosilicate (LYSO) scintillators—PMTs were used to design the DOI detector. Due to these PET designs, the spatial resolution of HRRT is as high as 2.5 mm full width at half-maximum (FWHM), which is still the highest resolution. Figure 2 shows an example of the image resolution of HRRT-PET.

**Figure 3:**
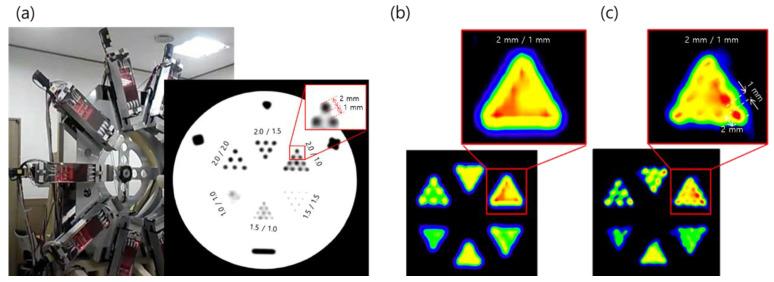
(**a**) Prototype high-resolution wobbling and zooming PET system developed by our group. (**b**) Images of the spatial phantom obtained via HRRT-PET and (**c**) by the prototype wobble-PET system. Reprinted with permission from Cho et al., 2019, IEEE [34].

Recently, our group proposed a novel PET system for high-resolution imaging and developed a prototype machine, as shown in Figure 3 [34]. The blurring of the PET image due to physical properties between the detectors and gamma photons that degrade the spatial resolution of PET can be measured as a point spread function or a line spread function (LSF) [35]. In the prototype system, LYSO scintillators with a 3.85 mm detector width—SiPMs were used to design the 1:1 coupling. We used the wobbling mode [36,37] with linear interpolation and reconstruction of the LSF deconvolution and achieved a substantially higher spatial resolution than conventional PET systems [34]. By combining LSF deconvolution reconstruction and wobbling sampling, we achieved a 1.56 mm FWHM transaxial resolution compared with 2.47 mm FWHM of HRRT, which was the highest among PET systems developed for both research and clinical applications. Ideally, the proposed PET system can resolve the submillimeter resolution currently available for small PET detectors using an oversampling technique.

### 2.3. PET–MRI Fusion Imaging

The hybrid system provides more clinically useful information than a stand-alone system [38,39]. Recently, the emerging PET–MRI fusion system is being actively researched and developed because it can overcome problems such as excessive radiation exposure and low soft-tissue contrast of CT with PET–CT system [6,40]. There are several types of combining PET and MRI into a hybrid system [21]. The technique of inserting a PET gantry inside the bore of the MRI scanner [41,42] or integrating the PET and MRI scanners in a single gantry [43,44] has the technical issues associated with the mutual interference between the two imaging modalities, including image artifacts related to the homogeneity of MRI fields in the presence of the PET detector, RF shielding for PET, and attenuation correction of PET data [45,46,47,48]. On the other hand, the tandem hybrid system in which PET and MR images are sequentially acquired by moving a patient bed between the two scanners could greatly reduce the mutual interference issues [49,50].

**Figure 4:**
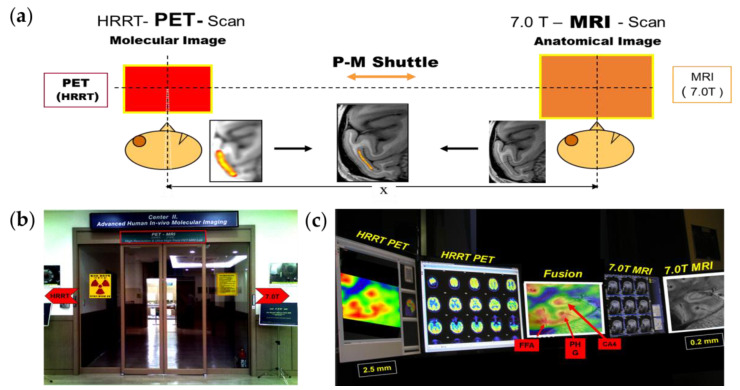
(**a**) Conceptual diagram of the high-resolution PET–MRI fusion imaging system and the developed PET–MRI system with HRRT and 7.0T MRI. (**b**) A gate of the PET–MRI fusion system using HRRT-PET and 7.0T MRI. The shuttle is visualized in the middle of the chamber. (**c**) Results are displayed on the PET–MRI console showing the PET images on the left and the MR images on the right. In the middle, a PET–MRI fusion image is shown. P-M; PET-MRI, FFA; fusiform face areas, PHG; parahippocampal gyrus, CA4; cornu ammonis 4. Reprinted with permission from Cho et al., 2008, JOHN WILEY AND SONS [49].

We developed a PET–MRI fusion system that combines HRRT-PET and 7.0T MRI for functional and molecular imaging with structural imaging for molecular imaging [49]. As we know, the spatial resolution of PET images compared to MRI is low. The resolution of MRI is approximately 1.5 × 1.5 × 4 mm^3^ for 1.5–3.0 T scanners and up to 80 × 80 × 200 µm^3^ for a UHF, 11.7T research MRI [51]. Conversely, the resolution of conventional whole-body PET is less than 5 mm, and the resolution of brain-dedicated PET is about 1.5 to 4 mm [34]. Therefore, to match the resolution mismatch of PET and MR images, we developed a PET–MRI fusion imaging system with a precision shuttle between 7.0T MRI and HRRT-PET, which resulted in image matching precision down to 0.05 mm by precision mechanical alignment. Although it is not as ideal as single-unit fusion PET–MRI, it is currently the most technologically feasible PET–MRI system. For the first time, it demonstrated precision PET–MRI fusion imaging, especially with high-resolution 7.0T MRI and HRRT-PET, by coupling the two with the shuttle concept, as shown in Figure 4.

### 2.4. Applications of PET–MRI

**Figure 5:**
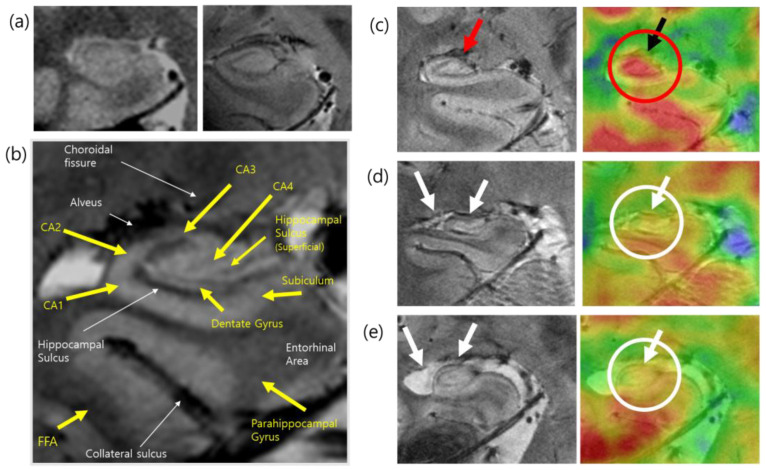
(**a**) Comparison of hippocampus imaging in 1.5T (left) and 7.0T (right) MRI. (**b**) Anatomical labeling of the hippocampus substructure in the 7.0T MR image. This research was originally published in JNM [52]. © SNMMI. (**c**) A 7.0T structural MRI and corresponding [^18^F]FDG PET–MRI fusion image of the hippocampus of healthy individuals (**d**) and (**e**) hippocampal atrophy and deformation in the 7.0T MR images and the corresponding glucose metabolism in the [^18^F]FDG PET–MRI of patients with AD. FFA; fusiform face areas, CA1-4; cornu ammonis 1-4. Reprinted with permission from Cho et al., 2014, Elsevier [13].

A fused image of the hippocampus is displayed in the middle of the display console. The hippocampus is an area of interest to many neuroscientists because it is related to memory and human cognition. Although the hippocampus is small, it contains several structurally important subregions that are closely connected to other brain regions. Using high-resolution 7.0T MRI, the subregions of the hippocampus can be structurally visualized and compared with those defined in previous postmortem studies. Cell density, microvessels, and small gaps in the hippocampal sulcus and dentate gyrus can be clearly visualized in MRI of the hippocampus. When we combined and fused the PET image with the high-resolution MRI with a precision of 0.05 mm, the functional and molecular imaging data could be precisely located at the desired anatomical location [52]. Because PET images alone have a spatial resolution that is insufficient to localize in the proper hippocampal subregions, PET–MRI fusion can provide accurate information for the successful localization and quantification of glucose metabolism in its small substructures, including the CA1, CA2, CA3, CA4, and dentate gyrus. Using this subregional mapping of glucose metabolism within hippocampal areas, some clinical cases of encephalitis were studied, and abnormal glucose metabolism was identified. Structural atrophy and deformation in hippocampal encephalitis showed a significant decrease in glucose uptake, especially in the CA4 and proper hippocampal regions. Glucose metabolism has also been studied and compared in the hippocampal subdivisions of patients with mild Alzheimer’s disease (AD) and healthy controls. High-resolution PET–MRI fusion scans were performed in nine patients with early-stage AD and ten healthy individuals [53]. MRI were acquired using a two-dimensional T2*-weighted gradient echo sequence with the following scan parameters: repetition time (TR) = 750 ms, echo time (TE) = 21 ms, flip angle (FA) = 30°, resolution = 0.2 × 0.2 × 0.2 mm^3^, imaging orientation = coronal, and the number of slices = 17, and the [^18^F]FDG PET images were obtained with a voxel size of 1.22 × 1.22 × 1.22 mm^3^. Patients with early-stage AD exhibited significantly lower glucose metabolism in the posterior CA2/3 region of the left hippocampal body than the healthy controls (Figure 5).

**Figure 6:**
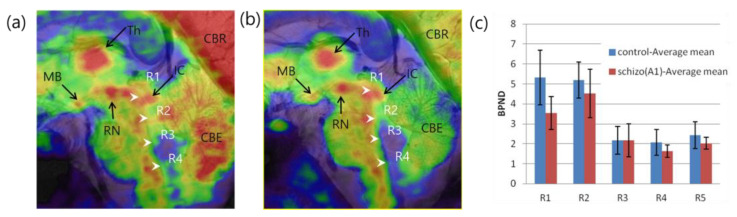
Raphe nuclei were identified in the brainstem by PET–MRI using (**a**) [^18^F]FDG and (**b**) [^11^C]DASB PET. (**c**) Group differences in the BP_ND_ of the SERT of healthy controls and schizophrenic patients observed by PET–MRI. Reprinted with permission from Son et al., 2012, Elsevier [54]. Th; thalamus, MB; mammillary body, RN; red nucleus, IC; inferior colliculus, R1-R4; raphe nuclei, CBR; cerebral cortex, CBE; cerebellum, schizo(A1); patients with acute schizophrenia.

The brainstem is another region where several nuclei modulate neuronal signals by controlling various neurotransmitters such as serotonin and dopamine (DA). Serotonin-producing nuclei and raphe nuclei are widely dispersed along the brainstem among neurotransmitters. One of the first high-resolution images of serotonergic raphe nuclei obtained using PET–MRI is shown in Figure 6 [54]. This high-resolution molecular imaging of glucose and serotonin transporter (SERT) is visible in PET–MRI fusion images. Kim et al. examined the relationship between self-transcendence and SERT availability in the brainstem raphe nuclei of 16 healthy individuals [55]. A high-resolution MRI of these nuclei was acquired using a T1-weighted three-dimensional magnetization-prepared rapid gradient echo sequence with the following scan parameters: TR = 751 ms, TE = 21 ms, FA = 30°, resolution = 0.18 × 0.18 × 1.5 mm^3^, and the number of slices = 17, and the HRRT-PET image of the SERT was obtained with a voxel size of 1.25 × 1.25 × 1.25 mm^3^. This study analyzed the total self-transcendence score, which showed a significant negative correlation with SERT binding potential (BP_ND_) in the caudal raphe. The subscale score for spiritual acceptance was significantly negatively correlated with SERT BP_ND_ in the median raphe nucleus.

## 3. Molecular Brain Imaging in Psychiatric Disorders

Molecular brain imaging studies have examined potential neurochemical biomarkers involved in various psychiatric disorders and have shown evidence of alterations in many other neurochemical systems in these diseases. Herein, we have reviewed the main findings across various neurochemical systems in the brainstem, hippocampus, and thalamus in the field of psychiatric disorders, including schizophrenia and major depressive disorder (MDD). Furthermore, the main findings in the striatum and globus pallidus were reviewed, considering the pathophysiology and symptomatology of these diseases. For these reviews, the investigators searched for clinical articles published between 1 January 2011 and 30 September 2021, in PubMed, using keywords such as “PET” AND “schizophrenia” OR “major depressive disorder” AND “serotonin” OR “dopamine” OR “glutamate” OR “norepinephrine” AND “brainstem” OR “thalamus” OR “hippocampus” OR “amygdala” OR “striatum”. These reviews included full-text articles showing the results of between-group comparisons in ROIs but excluded abstracts, methodological articles, review articles, duplicate articles, and conference proceedings. A total of 37 articles were identified in PubMed, and 15 articles met the inclusion criteria. Details of the radiotracers reviewed in this section are listed in Table 1.

### 3.1. Schizophrenia

Recent major findings in schizophrenia have been reported in all ROIs, excluding the brainstem, in the serotonergic and dopaminergic systems.

**Figure 7:**
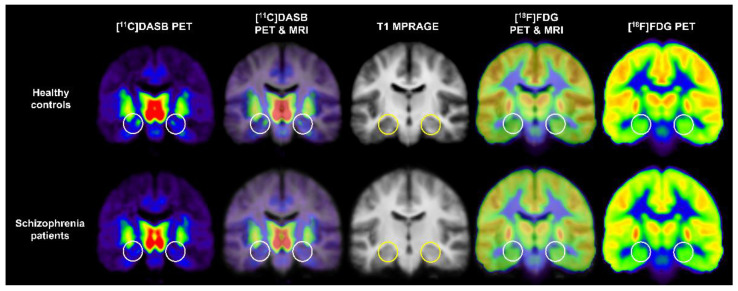
Mean [^11^C]DASB PET, [^18^F]FDG PET, T1 MRI, and PET–MRI fusion images of healthy controls and patients with schizophrenia. Circles indicate the location of the bilateral anterior hippocampus.

Two PET studies in patients with schizophrenia showed serotonergic and dopaminergic dysfunction in the hippocampus [60,67]. The first study by Kim et al. investigated the interregional correlation patterns between SERT availability and glucose metabolism using 7.0T MRI and HRRT-PET with [^11^C]DASB and [^18^F]FDG in 19 antipsychotic-free patients with schizophrenia and 18 healthy controls to observe abnormal functional connectivity in schizophrenia [60]. In particular, Kim et al. evaluated the differences in SERT availability and glucose metabolism in all brain regions between groups, including the anterior and posterior hippocampi, based on previous studies suggesting functional segregation in hippocampal subregions [72,73,74]. They reported decreased SERT availability in the anterior hippocampus in patients with schizophrenia compared with healthy controls but not in glucose metabolism at the threshold of two-tailed *p* < 0.01 [60]. Moreover, this study revealed no significant correlation between SERT availability and glucose metabolism in each group’s whole hippocampus or hippocampal subregions [60]. Kim et al. reported significant differences between groups in the correlations between SERT availability in the parietal and temporal cortices and glucose metabolism in the posterior cingulate cortex. These results suggest altered functional circuitry related to the posterior cingulate gyrus in the pathophysiology of schizophrenia. Figure 7 shows mean [^11^C]DASB PET, [^18^F]FDG PET, T1 MRI, and PET–MRI fusion images of healthy control subjects and patients with schizophrenia obtained in the PET study reported by Kim et al. [60].

The second study by Artiges et al. explored striatal and extrastriatal DA dysfunction in schizophrenia using HRRT-PET with [^11^C]PE2l in 21 male patients with chronic schizophrenia and 30 healthy male controls [67]. Artiges et al. reported an increase in DAT availability in the hippocampus in schizophrenia and significant positive correlations of DAT availability in the hippocampus with hallucinations and unusual thought content in schizophrenia. This study also showed that patients with schizophrenia have a higher DAT availability in the left thalamus. Artiges et al. reported that the high resolution and sensitivity of HRRT-PET enabled the evaluation of increased DAT availability in the hippocampus of patients with schizophrenia. Furthermore, this study demonstrated that these results were consistent with previous PET studies that suggested presynaptic DA hyperactivity in schizophrenia and that striatal and extrastriatal DA dysfunction are involved in positive psychotic symptoms. PDE10A is an enzyme present in DA neurons that degrades intracellular secondary messengers triggered by DA signaling. PDE10A inhibitors are of interest in clinical studies and the pharmaceutical industry related to psychiatric disorders because they have an antipsychotic-like effect in preclinical studies [75,76,77,78,79]. With the recent development of [^11^C]IMA107, a selective PET radiotracer for PDE10A, Marques et al. investigated PDE10A availability in 12 patients with chronic schizophrenia and 12 healthy controls using [^11^C]IMA107 PET [71]. This study revealed that PDE10A availability in the thalamus did not differ between groups and was not significantly correlated with the severity of psychotic symptoms or antipsychotic dosage. This study demonstrated that patients with schizophrenia had normal PDE10A availability in the thalamus, which is consistent with the results of previous preclinical studies showing no change in PDE10A availability in animal models of schizophrenia [75,77,78,80,81,82].

Most PET studies on schizophrenia reviewed in this study revealed alterations in the serotonergic and dopaminergic systems of the striatum [59,63,64,67,71] and globus pallidus [71]. A previous PET study examined the availability of 5-HT_6_ receptors in the striatum and 5-HT_2A_ receptors in the cortex following treatment with olanzapine, risperidone, aripiprazole, and quetiapine in nine male patients with schizophrenia and nine healthy male controls using PET with [^11^C]GSK215083 [59]. All patients underwent [^11^C]GSK215083 PET scans at the presumed steady-state trough level (trough scan) and peak serum level (peak scan) after seven days of antipsychotic treatment. This study revealed that patients treated with olanzapine showed lower availability of 5-HT_6_ and 5-HT_2A_ receptors (range: 53–95%) in the ventral striatum, caudate, putamen, and frontal cortex at both trough and peak scans than healthy controls. Furthermore, patients treated with quetiapine had a lower availability of 5-HT_6_ receptors in the putamen at the trough scan (34%) and peak scan (45%) relative to controls. This study suggests that different antipsychotic treatments alter 5-HT_6_ and 5-HT_2A_ availability.

Caravaggio et al. estimated the D_2/3_ receptor BP_ND_ before and after DA depletion in three male patients with schizophrenia treated with olanzapine and ten healthy controls (six males and four females) using [^11^C]PHNO PET to explore the changes in endogenous DA in the striatum of medicated schizophrenia [63]. This study revealed that patients treated with olanzapine showed greater ΔBP_ND_ (i.e., the fractional increase in D_2/3_ receptor BP_ND_ after DA depletion) in the caudate and putamen than healthy controls. These findings suggest that relapse in clinical symptoms can occur when antipsychotic medications are administered. A PET study by Veselinović et al. investigated the relationship between cognitive function and dopaminergic transmission in the striatum in 15 medication-free patients with schizophrenia and 11 controls, using PET with [^18^F]fallypride and neurocognitive assessment [64]. In this study, cognitive function was evaluated in all participants using the trail making test (TMT), measuring complex visual scanning with a motor component, motor speed, and agility in parts A and B [83], digit-symbol-substitution task (DSST) quantifying the speed of mental processing [84], verbal fluency task (“Regensburger Wortflüssigkeits-test”) assessing phonemic and semantic fluency and the ability to change categories [85], and Letter-Number Span evaluating working memory performance [86]. This PET study revealed an approximately 10% higher D_2/3_ receptor availability in the caudate and putamen of patients than those in controls, but the difference was not statistically significant [64].

Furthermore, cognitive performance of the TMT in individual patients was significantly negatively correlated with D_2/3_ receptor availability in both the caudate and putamen. Moreover, cognitive performance in the DSST in each patient was significantly positively associated with D_2/3_ receptor availability in the caudate nucleus. These results corroborated that D_2/3_ receptor signaling was more involved in specific cognitive functions in patients with schizophrenia than in controls and suggested that relatively lower basal occupancy by endogenous DA in these patients favors better sparing of cognitive function.

In addition to the increased availability of DAT in the hippocampus of patients with schizophrenia, Artiges et al. reported that the availability of DAT in the left caudate head/nucleus accumbens and putamen increased in patients with schizophrenia compared with controls [67]. Furthermore, DAT availability in the hippocampus, putamen, and globus pallidus was significantly correlated with hallucinations and suspiciousness/persecution.

In addition to the thalamus, as mentioned above, a PET study by Marques et al. confirmed the differences between groups in PDE10A availability in the striatum [71]. In this study, no significant changes in the availability of PDE10A were found in the caudate, putamen, and globus pallidus in patients with schizophrenia compared to controls (% change in patients, −1–6%). In addition, the level of PDE10A binding in these regions was not significantly related to the severity of psychotic symptoms. Based on a study of intracellular signaling pathways demonstrating the effect of PDE10A inhibitors on overall signaling in the therapeutic direction of schizophrenia [76] and several studies demonstrating that PDE10A inhibitors produce behavioral effects that predict antipsychotic activity, similar to D_2_ antagonists [75,77,78,80,81,82], this study suggests that intracellular signaling pathways affected by PDE10A inhibitors in schizophrenia should be considered compensatory pathways, rather than pathological mediators.

### 3.2. Major Depressive Disorder

Recent major findings of ROIs, excluding the brainstem in MDD, have emerged in various neurotransmitter systems such as serotonin, DA, and norepinephrine.

A PET study by Tiger et al. investigated the binding of 5-HT_1B_ receptors in brain regions associated with MDD pathophysiology in ten drug-free recurrent patients and 10 controls using PET with [^11^C]AZ10419369 [57]. Tiger et al. reported lower 5-HT_1B_ receptor BP_ND_ in the hippocampus in patients with recurrent MDD than that in controls (32% between-group difference). This study suggested that the result was in line with the decreased binding of the 5-HT_1A_ receptor in this region, as reported in previous MDD studies using [^11^C]WAY100635 PET [56]. However, this study further suggested that additional observations are needed for a more detailed understanding of serotonergic innervation in the hippocampus, as 5-HT_1A_ and 5-HT_1B_ receptors are functionally similar, but postsynaptic and presynaptic autoreceptors, respectively, and in different cortical layers [57].

Two PET studies investigated the NET levels in patients with MDD and healthy controls using (S,S)-[^18^F]FMeNER-D2 PET [69,70]. The first study by Moriguchi et al. was conducted on 19 patients with MDD and 19 healthy controls to evaluate the availability of NET and its role in the clinical symptoms of MDD [69]. This study reported that patients with MDD had higher NET availability in the thalamus and thalamic subregions anatomically connected to the prefrontal cortex than controls. Furthermore, Moriguchi et al. found that NET availability in the thalamus was negatively correlated with reaction time in the TMT(A) in patients with MDD. This study suggests that increased norepinephrine transmission in patients with MDD could be associated with preserved visual attention, as estimated by reaction time in the TMT(A). The second study by Arakawa et al. investigated NET occupancy of clinically relevant doses of venlafaxine extended-release (ER) in 12 patients with MDD who responded to venlafaxine ER and nine healthy controls [70]. Arakawa et al. reported that NET BP_ND_ in the thalamus was significantly lower in patients treated with 150~300 mg/d venlafaxine than in controls. However, it was not significant in patients treated with 37.5~75 mg/d venlafaxine, suggesting that the NET BP_ND_ in the thalamus was inversely related to the dose of venlafaxine ER. Furthermore, the NET occupancy increased in a dose- and plasma-concentration-dependent manner, although no significant differences were observed above 150 mg/d. This study revealed that clinically relevant doses of venlafaxine ER block NETs formation in the brains of patients with MDD. A PET study by Yeh et al. reported altered SERT availability in the thalamus of patients with MDD [61]. This study examined the role of SERT in MDD and suicidal behavior in 17 antidepressant-naïve patients with MDD (i.e., eight depressed suicidal and nine depressed non-suicidal patients) and 17 healthy controls using PET with 4-[^18^F]-ADAM. This study found that SERT availability in the thalamus was significantly reduced in the MDD and depressed suicidal groups. However, this study also reported that the reduced availability of SERT in the thalamus in the depressed suicidal group was inconsistent with the results of a previous study by Ney et al. [87]. In most of the PET studies reviewed, patients with MDD showed serotonergic or dopaminergic dysfunction in the striatum [58,61,62,63,64,65,66].

In the serotonergic system, a previous PET study investigated the availability of the 5-HT_1B_ receptor in the ventral striatum/ventral pallidum in ten patients with MDD in a current major depressive episode and 10 control individuals using HRRT-PET with [^11^C]P943 [58]. This study reported lower availability of 5-HT_1B_ receptors in the ventral striatum/ventral pallidum in patients with MDD than in controls. This study demonstrated results that were consistent with those of previous preclinical and postmortem studies [88,89,90] and suggested that abnormal 5-HT_1B_ heteroreceptor function may be associated with dysfunctional reward signaling in the striatum through interactions with the DA, γ-aminobutyric acid, or glutamate systems [58]. In addition to the reduced availability of SERT in the thalamus in patients with MDD, Yeh et al. reported that SERT availability in the striatum was decreased in all patients with MDD and suicidal patients with depression [61]. Based on previous findings that the striatum and corticobasal ganglia pathways play an important role in the neuropathology of affective disorders and subsequent suicidal behaviors [91] and are associated with reward prediction involved in decision-making [92], one study suggested that the reduced availability of SERT in the striatum in patients with MDD and depression may contribute to the development of suicidal action [61].

Hamilton et al. investigated deficits in the stimulation of striatal DA receptors in MDD by decrementing the propagation of information along the cortico-striatal-pallido-thalamic (CSPT) circuit [62]. In this study, 16 MDD patients and 14 healthy controls were scanned using [^11^C]raclopride PET and functional MRI. Hamilton et al. found an increase in the D_2_ receptor BP_ND_ in both the dorsal striatum and ventral striatum in patients with MDD compared to controls. This study reported significant negative correlations between altered D_2_ receptor BP_ND_ in the dorsal striatum and ventral striatum and connectivity in each default-mode network and salience network. Hamilton et al. suggested that the reduced striatal D_2_ receptor BP_ND_ in MDD could be partly associated with the failure of information transfer to the CSPT circuit in the pathophysiology of this disorder.

Furthermore, two PET studies reported alterations in DAT levels in patients with MDD [65,66]. Pizzagalli et al. investigated 25 patients with MDD and 23 healthy controls using [^11^C]-altropane PET and reported that patients with MDD had lower availability of DAT in the bilateral putamen than controls [65]. This study suggests that MDD is characterized by reduced DAT expression in the striatal region. Moriya et al. estimated DAT availability in 11 geriatric patients with severe MDD and 27 healthy controls using PET with [^18^F]FE-PE2I, assuming that anhedonia, a clinical feature of geriatric patients with MDD, is associated with reduced DA neurotransmission in the reward system [66]. Moriya et al. reported that geriatric patients with severe MDD had significantly lower DAT availability in the putamen than in the healthy controls, suggesting a link between dopaminergic neuronal dysfunction and dysregulation of the reward system.

## 4. PET–MRI Applications in Psychiatric Disorders

For this section, we searched for clinical articles published between 1 January 2015 and 30 September 2021 in PubMed using keywords such as “PET–MRI” AND “psychiatric disorders“ OR “schizophrenia” OR “major depressive disorder”. This review includes full-text articles on the use of PET–MRI fusion imaging techniques in psychiatric disorders, including schizophrenia and MDD, but excludes abstracts, methodological articles, review articles, duplicate articles, and conference proceedings. Two articles were identified in PubMed, and one article met the inclusion criteria. Details of the radiotracers reviewed in this section are listed in Table 1.

PET–MRI studies have rarely focused on patients that have psychiatric disorders. A novel fusion imaging tool, the PET–MRI-electroencephalography (EEG) system, was used in a study on schizophrenia [68]. To observe the changes in the dopaminergic system in schizophrenia, this study was performed using a whole-body mMR Biograph PET–MRI scanner (Siemens AG Healthcare, Erlangen, Germany) with [^18^F]-FDOPA in 12 patients with schizophrenia and 13 healthy controls. In this study, patients with schizophrenia showed increased DA synthesis capacity in the nucleus accumbens and functional limbic region (which largely overlaps with the anatomical region of the nucleus accumbens) compared to healthy controls. These results suggest that PET–MRI fusion imaging is replicable in detecting significant group differences in the dopaminergic system and provides excellent anatomical-functional coregistration in some small regions, such as the nucleus accumbens and functional limbic region.

Moreover, this study was conducted using a PET–MRI–EEG system with [^11^C]ABP688 during a mismatch negativity (MMN) task [93] in a schizophrenic patient and a healthy control to explore the feasibility of the trimodal acquisition protocol. In this experiment, schizophrenia patients had significantly reduced functional connectivity in both auditory and salience networks than healthy control. During the MMN task, the average [^11^C]ABP688 BP_ND_ values exhibited changes in the precuneus, posterior cingulum, hippocampus, parahippocampus, nucleus accumbens, and middle frontal cortex, and inferior frontal cortex in a patient with schizophrenia compared with healthy control. In addition, the loudness dependence of auditory evoked potential EEG data showed a significant group difference at a single trial level during the MMN task. These results suggest the potential use of the PET–MRI–EEG system as a fusion imaging tool for detecting biomarkers of schizophrenia. However, this is the result from a single schizophrenia patient, and this study suggests that these results need to be replicated with a much larger sample.

## 5. Conclusions

The brain is the most complex system in the functional domain. Within a few micrometers to millimeters, brain function differs. As shown in recent studies of psychiatric disorders, various regions of the brain, including the striatum, thalamus, and hippocampus, are involved in the alterations of neurotransmitters. Subdividing these regions into high-resolution regions can help differentiate brain diseases, especially psychiatric disorders.

Until now, a majority of PET radioligands targeting neurotransmitter receptors, transporters, and enzymes are labeled with carbon-11 [94,95]. Therefore, in the realm of psychiatry, the routine clinical use of radioligand PET imaging has limitations due to the requirement of an onsite cyclotron facility. However, PET research with 11-C radioligands probing neurotransmitter receptors and transporters has significantly contributed to unraveling the complex pathophysiology of psychiatric disorders such as schizophrenia (DA hypothesis) and depression (serotonin and catecholamine hypothesis) [94,96]. Therefore, PET imaging studies using 11-C radioligands in psychiatric disorders were mainly reviewed in this paper. The research studies can also play a significant role in the new drug development processes in the early phase using proof of concept trials [97] and in the late development phase using drug occupancy studies [98].

Regarding the hybrid PET–MRI system, high spatial resolution is particularly important in brain imaging, where structures and functions differ by a few millimeters. Therefore, considering that conventional whole-body PET has a resolution of <5 mm, it is essential to improve the resolution of PET to combine the different information between the two modalities. Achieving a spatial resolution of a PET image of <1 mm is required to make the PET–MRI fusion system truly useful for research and clinical applications. A new PET–MRI fusion system with “wobble mode” PET in conjunction with 7.0T is another future close-coupled PET–MRI fusion system with which submillimeter PET–MRI imaging can be achieved.

Over the last several decades, we have witnessed numerous medical imaging devices and improvements in their performance, which has led to immense progress in medical sciences, especially in conjunction with pharmaceuticals. As representative examples, medical imaging systems, specifically CT, MRI, and PET, have changed the basic methodology for diagnosing diseases. In particular, the emerging PET–MRI fusion system is an advanced medical imaging technique that can overcome problems such as excessive radiation exposure and low soft-tissue contrast in CT with fusion PET–CT devices, which are currently the most widely used in clinical practice. Hybrid PET–MR imaging is expected to contribute more accurate and useful information than PET–CT for the development of new pharmaceuticals. When measuring the occupancy of pharmaceuticals for the specific target neuroreceptor, delineating the anatomical structures with high resolution will improve the accuracy of measurements and the specificity of the binding region and, therefore, the care provided.

## Figures and Tables

**Table 1:** Tracer targets and radiotracers reviewed in Section 3 and Section 4.

Tracer target	Radiotracer	Name: Synonyms	Study
Serotonin 5-HT_1A_ receptor	[^11^C]WAY100635	*N*-[2-[4-(2-methoxyphenyl)-1-piperazinyl]ethyl]-*N*-(2-pyridinyl)cyclohexanecarboxamide trihydrochloride	Anisman et al. [56]
Serotonin 5-HT_1B_ receptor	[^11^C]AZ10419369	5-methyl-8-(4-[^11^C]methyl-piperazin-1-yl)-4-oxo-4*H*-chromene-2-carboxylic acid (4-morpholin-4-yl-phenyl)-amide	Tiger et al. [57]
	[^11^C]P943	*R*-1-[4-(2-methoxy-isopropyl)-phenyl]-3-[2-(4-methyl-piperazin-1-yl) benzyl]-pyrrolidin-2-one	Murrough et al. [58]
Serotonin 5-HT_6_ receptor	[^11^C]GSK215083	[^11^C]-[*N*-methyl]3-[(3-fluorophenyl)sulfonyl]-8-(4-methyl-1-piperazi nyl)quinoline	Radhakrishnan et al. [59]
SERT	[^11^C]DASB	[^11^C]-3-amino-4-(2-dimethylaminomethyl-phenylsulfanyl)- benzonitrile	Kim et al. [60]
	4-[^18^F]-ADAM	N,N-dimethyl-2-(2-amino-4-[^18^F]fluorophenylthio)benzylamine	Yeh et al. [61]
DA D_2_ receptor	[^11^C]raclopride	3,5-dichloro-N-[[(2S)-1-ethylpyrrolidin-2-yl]methyl]-2-hydroxy-6-[^11^C]methoxy-benzamide	Hamilton et al. [62]
DA D_2/3_ receptor	[^11^C]PHNO	[^11^C]-(+)-4-propyl-9-hydroxynaphthoxazine	Caravaggio et al. [63]
	[^18^F]fallypride	5-(3-[^18^F]fluoropropyl)-2,3-dimethoxy-N-[(2S)-1-prop-2-enylpyrrolidin-2-yl]methyl]benzamide	Veselinović et al. [64]
DA transporter (DAT)	[^11^C]-altropane	*2β*-carbomethoxy-*3β*-(4-fluorophenyl)-N-((E)-3-iodo-prop-2-enyl)tropane	Pizzagalli et al. [65]
	[^18^F]FE-PE2I	N-(3-iodoprop-2E-enyl)-2β-carbo-[^18^F]fluoroethoxy-3β-[4-methylphenyl]-nortropane	Moriya et al. [66]
	[^11^C]PE2l	[^11^C]N-(3-iodoprop-2E-enyl)-2β-carbomethoxy-3β-(4-methylphenyl)nortropane	Artiges et al. [67]
DA synthesis capacity	[^18^F]-FDOPA	[^18^F]-6-L-fluoro-L-3,4-dihydroxyphenylalanine	Guerra et al. [68]
Metabotropic glutamate receptor 5	[^11^C]ABP688	3-(6-methyl-pyridin-2-ylethynyl)-cyclohex-2-enone-O-[^11^C]-methyl-oxime	Guerra et al. [68]
Norepinephrine transporter (NET)	(S,S)-[^18^F]FMeNER-D2	(S,S)-2-(α-(2-[^18^F]fluoro[^2^ H_2_]methoxyphenoxy) benzyl)morpholine	Moriguchi et al. [69] Arakawa et al. [70]
Phosphodiesterase 10A (PDE10A)	[^11^C]IMA107	5-[(3*R*)-3-fluoropyrrolidin-1-yl]-*N*-[^11^C]methyl-2-(3-methylquinoxalin-2-yl)-N-tetrahydropyran-4-yl-pyrazolo[1,5-α]pyrimidin-7-amine	Marques et al. [71]
Glucose metabolism	[^18^F]FDG	2-deoxy-2-[^18^F]fluoro-D-glucose	Kim et al. [60]

## Data Availability

Data sharing not applicable.
